# Paget disease of the vulva an analysis of 24 cases

**DOI:** 10.1097/MD.0000000000017018

**Published:** 2019-10-11

**Authors:** Rosalia Maria Rita Loiacono, Paola Traversi, Maria Assunta Deliso, Giulio Gargano, Anila Kardhashi, Roberta Francescato, Vera Loizzi, Marta Spinelli, Serena Lovascio, Maura Fiorito, Leonardo Resta, Ondina Popescu, Ettore Cicinelli, Gennaro Cormio

**Affiliations:** a Gynecologic Oncology Unit, IRCCS Istituto Tumori “Giovanni Paolo II”; b Department of Obstetrics and Gynecology; c Department of Pathology, University of Bari; d Department of Pathology, IRCCS Istituto Tumori “Giovanni Paolo II,” Bari, Italy.

**Keywords:** Paget disease of the vulva, surgery, vulvar cancer

## Abstract

Paget's disease can arise in the breast (mammary Paget disease) or in other locations (extramammary Paget disease) such as anogenital skin in both males and females (Paget disease of the vulva [PDV]). Underlying adenocarcinoma can be found in some cases. This study aims to report clinical aspects, surgical procedures, outcomes, and recurrences of patients with PDV.

A retrospective chart review was conducted on patients with pathologically confirmed diagnosis of PDV managed at the Department of Obstetrics and Gynecology, University of Bari, and the “Giovanni Paolo II” National Cancer Institute in Bari, between 1998 and 2018.

Records of 24 cases of PDV were examined. Median age of the patients at diagnosis was 69.3 (range 38–84), diagnosis of synchronous cancer was made in 2 cases and in 2 other cases of metachronous disease. Three patients had previously been diagnosed with other oncological diseases. All patients underwent surgery including wide local excision (6), simple vulvectomy (8), and extended vulvectomy (10). Lymphadenectomy was performed in 2 cases and reconstructions with advancement flaps in 7 cases. Four patients were found to have invasive disease and 1 had inguinal node involvement. Positive margins were found in 11 patients. Wound dehiscence and urethral stenosis were found in 4 and 1 case each. Eight recurrences (33.33%) were observed, regardless of positive surgical margins.

PDV has a low rate of malignancy but a high rate of recurrence. It should be diagnosed early to avoid repeated surgery and to reduce symptoms and morbidity.

## 1 Introduction

Paget's disease can arise in the breast (mammary Paget disease [MPD]) or in other locations (extramammary Paget disease [EMPD]) such as the anogenital skin in females (Paget disease of the vulva [PDV]).

PDV is an uncommon pathologic condition^[1–8]^ accounting for from 1% to 2% of all tumors of the female genital area.^[3,9]^ The estimated incidence in Europe is about 0.7 cases per 100,000 women.^[2,4]^ It is thought to originate from adnexal structures, such as apocrine glands, multipotent stem cells in the epidermal basal layer or infundibular stem cells of the hair follicle.^[2,5–8]^

In 2001, Wilkinson et al subdivided vulvar Paget disease into primary and secondary disease.^[3,14,15]^

Primary (cutaneous) Paget disease is defined as an intraepithelial adenocarcinoma arising within the epidermis and extending into the contiguous epithelium of skin appendages. It is further classified into 3 subtypes: type 1a, intraepithelial Paget disease, representing 75% of cases; type 1b, intraepithelial Paget disease with invasion, representing16% of cases; and type 1c, intraepithelial Paget disease with an underlying adenocarcinoma of a skin appendage or a subcutaneous gland, with 9% of incidence.^[3,11]^

Secondary (noncutaneous) Paget disease is considered as an epidermotropic metastasis originating from an underlying malignancy of the gastrointestinal tract, of the urogenital tract, or of an adenocarcinoma originating elsewhere.^[3,11]^

The presence of Paget cells characterizes EMPD. These cells have prominent pale cytoplasm and a prominent central nucleolus, and are distributed throughout the epithelium, either as single cells or clusters with variable extent. The cytoplasm contains diastase resistant PAS positive material^[1]^ Paget cells can be visualized using hematoxylin and eosin staining. The scattered Paget cells are diagnostic, but they are interspersed within the normal epithelium and it can be difficult to detect them at times.^[2]^ Invasion is characterized by the presence of dyscohesive neoplastic Paget cells infiltrating the underlying dermis or submucosa.^[2,16]^

The degree of invasion beyond the basement membrane is categorized into 3 levels:

(1) in situ in the epidermidis;
(2) microinvasion to the papillary dermis;
(3) deep invasion to the reticular dermis or subcutaneous tissue.^[3,16]^

Immunohistochemistry is mandatory for correct diagnosis of Paget disease. Paget cells express Cytokeratin 7 (CK7), Cytokeratin 5.2, carcinoembryonic antigen, gross cystic disease fluid protein 15, human epidermal growth factor receptor 2, carbohydrate antigen 125, and androgen receptors, but do not express markers of squamous cell differentiation, such as p63 and p40, and melanocyte markers, such as Melan-A, Human Melanoma Black-45, or S100 proteins. p53 protein overexpression in the intraepidermal component is associated with invasion. Uroplakin-III, CK7 and CK20 and GATA3 gene are expressed in Paget disease secondary to urothelial carcinoma.^[1,2,9]^ CK20, Homeobox protein CDX-2, and Mucin 2 positivity (but not CK7) might indicate an underlying anorectal adenocarcinoma.^[1,2,9]^

EMPD is also associated with other malignancies, such as malignancies outside the vulva from 2% to 54% of cases. These may include breast, intestinal, and urologic malignancies. For this reason, in patients with biopsy-confirmed Paget disease, there should be further evaluation of the breast, genitourinary tract, and gastrointestinal tract.^[2,4,15,17]^

There are no pathognomonic symptoms or specific clinical aspects. Irritation, itching, burning, and vulvar pain are the most frequent symptoms. Sometimes vulvar paget disease (VPD) can be asymptomatic.^[2–4]^ Often VPD looks like a red eczematoid pruritic lesion, sometimes as an erythematous plaque with typical white scaling. The plaque may be ulcerated and crusted with a papillomatous surface.^[1,2]^

The clinical picture is usually aspecific^[1–4,17]^ and for this reason diagnosis is usually made in very extensive disease.

Surgery plays a major role in the treatment of PDV and the occurrence of positive margins is very common.^[3–6,8]^ However, there is no clear relationship between recurrent disease and the presence of positive margins or invasive disease.^[3–6,8]^

We retrospectively review our experience with PDV in order to evaluate clinical presentation, surgical treatment, and prognostic factors for recurrence and survival.

## 2 Materials and methods

A retrospective chart review was conducted on all patients with PDV managed at the Department of Obstetrics and Gynecology, of the University of Bari, and at the Gynecologic Oncology Unit, “Giovanni Paolo II” National Cancer Institute in Bari, Italy between 1998 and 2018. Institutional Review Board approval was obtained with a waiver of informed consent. Medical records were reviewed for demographic information, clinical data, pathologic findings, treatment modalities, and outcomes. At the time of diagnosis, all patients received intensive staging with pelvic examination, transvaginal ultrasound, PAP smear, chest X-ray, mammography, cystoscopy, and rectoscopy to rule out other sites of disease. All patients were operated by a gynecologic oncologist. Pathologic diagnosis of PDV was confirmed by 2 gynecologic pathologists (LR and OP). Invasive Paget's disease was defined as disease extending for at least 1 mm beyond the basement membrane. A positive margin was defined as Paget's cells within 1 mm of the surgical margin. No secondary surgical procedure or other adjuvant treatment (radiotherapy or medical treatment) were administered in such cases. Inguinal radiation was administered only in cases of nodal metastasis.

Physical examination was scheduled every 6 months, with biopsy in case of suspicious of recurrent disease. The follow-up period was defined as the time between initial Paget's disease diagnosis and the date of the last contact.

Descriptive statistics were used to summarize the patient demographic and clinical characteristics. Fisher exact test was used to compare patients with recurrence to those without recurrence, with respect to types of primary treatment received and margin status among those patients with primary surgery.

## 3 Results

Twenty-four patients were identified. The median age at diagnosis was 69.3 years (range 38–84 years). All were Caucasian.

The most common presenting symptoms were itching, burning, and pruritis associated with vulvar lesions in 5 patients, pain associated with pruritus in 3 patients, 1 patient complained of only itching another only vulvar pruritis. In 14 records, the symptoms presented by the patients were not reported (Table 1).

**Table 1:**
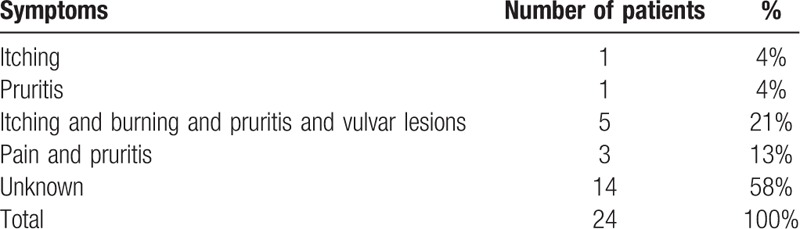
Symptoms.

Median duration of symptoms before diagnosis was 33.6 months (range 12–72 months). Twenty-three patients (96%) were primarily diagnosed and managed by a gynecologist, and 1 by a dermatologist (4%).

Two patients had medical treatment with imiquimod and 5 fluorouracil before surgery without any benefit.

All patients underwent surgery, including wide local excision 6 (25%), simple vulvectomy 8 (33%), and extended vulvectomy 10 (42%). Lymphadenectomy was performed in 2 cases (8%) and reconstructions with advancement flaps in 7 cases (29%). Wound dehiscence and urethral stenosis were found in 4 cases (17%) and 1 case (4%), respectively and were all managed conservatively (Table 2). The median operative time was 130 minutes (range 35–230 minutes) and the median length of hospital stay was 7.8 day (range 3–16). No patient had blood transfusion during or after surgery.

**Table 2:**
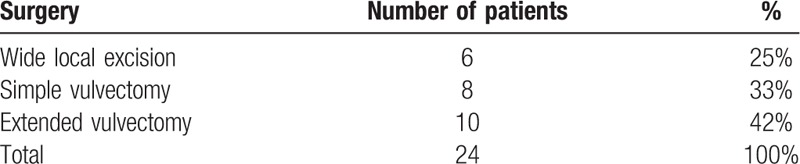
Surgical data.

The median tumor area was 48.9 cm^2^ (range 2.5–143 cm^2^). At pathologic examination 4 patients (16.67%) were found to have invasive disease and 1 of the 2 patients with invasive disease who had lymphadenectomy was found to have a single inguinal node involvement (4.17%). Margin status was available for all patients and positive margins were found in 12 patients (50%). The presence of positive margins was neither related to the extent of surgery (5 among patients with extended vulvectomies versus 7 among patients with simple vulvectomy) nor to the presence of invasive disease (50% of positive margins in patients with invasive disease and 50% in patients without invasive disease). No patient received adjuvant treatment with radiotherapy after primary surgery.

Diagnosis of synchronous cancer was made in 2 cases (vulvar squamous carcinoma, melanoma) and of metachronous cancer in 2 other cases (adenocarcinoma of the ampulla of Vater, 1 patient had endometrioid adenocarcinoma and urothelial carcinoma). Two patients had previously been diagnosed with breast cancer, 1 with breast cancer and ovarian cancer, 1 with bilinear myelodysplastic syndrome, and 1 with endometrioid adenocarcinoma (Table 3).

**Table 3:**
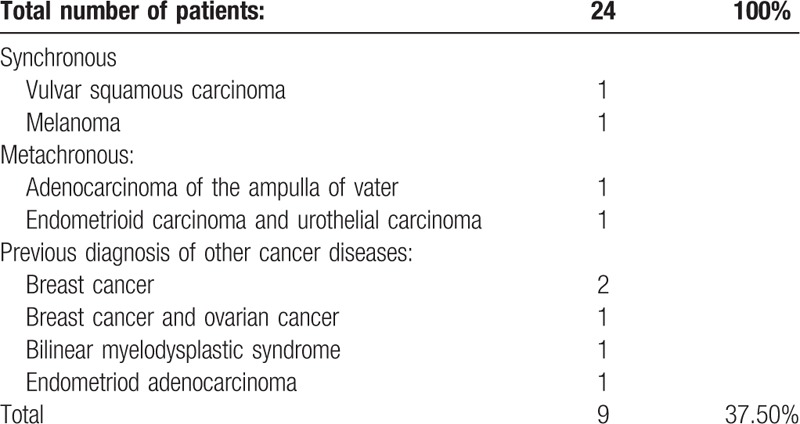
Synchronous, metachronous cancer and previous diagnosis of other cancer diseases.

Median follow-up was 39 months, ranging from 1 to 240 months. One patient was lost at follow-up. Eight recurrences (33%) were observed, regardless of histological type or positive surgical margins. In fact, in 6 cases of recurrence the margins were not affected by the disease as compared to 12 cases in patients with positive margins (17%). In all but 1 case, recurrence remained noninvasive. Four recurrences (50%) occurred in patients with urothelial carcinoma and metachronous carcinoma of the papilla of Vater, with synchronous melanoma, and with previous breast and ovarian carcinoma. In all cases of recurrence surgery was the only treatment modality applied with complete resection of the lesion in 100% of cases.

To date, 13 patients are free of Paget disease. Of these, 1 is affected by squamous cell carcinoma of the vulva and another 1 by bilinear myelodysplastic syndrome. Ten patients at follow-up are dead, 5 due to nononcological diseases. The cause of death of the other 5 is respectively: melanoma, adenocarcinoma of the ampulla of Vater, ovarian cancer, and urothelial carcinoma. Only 1 died because of invasive PDV.

## 4 Discussion

Clinicopathologic information regarding PDV is limited and controversial due to the rarity of the disease.^[2,5–8]^

Since in the initial phase it does not present characteristic clinical features, there can be delayed diagnosis or misdiagnosis.^[4–6]^ The time between the first symptoms and the correct diagnosis of PDV can vary from a few to several months.^[3–6]^ In our patients, the date of onset of the vulvar symptoms precedes from 1 to 6 years the biopsy and the diagnosis of Paget's disease.

Differential diagnoses may include cutaneous candidiasis, tinea cruris, seborrheic dermatitis, and psoriasis, Bowen disease, or melanoma (pigmented variant of EMPD),^[5]^ and for this reason VPD has to be diagnosed only with vulvar biopsy.

In our series, 20 patients (83.33%) were affected by primary Paget disease, similar to the majority of series in the literature.^[3,4,6,8,15–17]^

The importance of immunohistochemistry for the diagnosis of EMPD has now been reiterated in all studies. It is also important to make possible differential diagnoses with other oncological diseases of the vulva, such as melanoma and squamous carcinoma. Finally, with immunohistochemistry we can highlight secondary forms of Paget's disease that underlie urinary and gastrointestinal tract carcinomas. This is also confirmed by our study despite its limitations; in fact, immunohistochemistry in our study is available in only 11 cases out of 24.^[1,2,5–9]^

In our series, 4 patients were found to have invasive disease and this finding is similar to that reported in other large series.^[2,4,6,8,15–17]^

Surgery is still considered the gold standard of treatment for patients with PDV,^[7]^ and it is a protective factor for overall survival.^[8]^ In our study all patients underwent surgery.

In our series, primary management was medical in the only patient managed by a dermatologist, whereas when diagnosis was made by a gynecologist, surgical treatment was the preferred initial management.^[7,8]^

Due to the extent of disease at the time of diagnosis, 7 patients required plastic reconstructive procedures after surgical resection and this data is higher than that reported in other series. Different reconstructive techniques may be applied, but in our centre, we preferred V-Y advancement flaps.

The recurrence rate was high (33%) regardless of histological type or positive surgical margins. In fact, in 6 cases of recurrence the margins were not affected by the disease. In all cases of recurrence, the patients underwent surgery again. In the 2 cases of second and in the only case of third recurrence, surgery was still the therapy carried out. This finding is in line with the mainly surgical treatment of relapses which is reported in the literature.^[3–6,8]^

Our retrospective study confirms that in case of recurrence the disease rarely become invasive, so once the presence of underlying or co-existent malignancy is excluded, we could opt for the use of Topical 5% imiquimod cream, now widely considered effective for high grade vulvar squamous cell intraepithelial lesions.^[20,21]^

It is interesting to note that 4 recurrences occurred in patients with urothelial carcinoma and metachronous carcinoma of the papilla of Vater, with synchronous melanoma, and with a previous breast and ovarian carcinoma.

Five of the 10 dead patients died due to non-oncological reasons, suggesting that Paget disease is not a fatal disease. Only 1 patient had a very aggressive disease leading to sudden system diffusion and death. In fact, the course is unpredictable. In situ forms often do not progress for many years, but in cases of invasion of the dermis the prognosis also becomes rapidly unfavourable.^[3,9]^

In the remaining 4 patients death was due to associated cancer: melanoma, adenocarcinoma of the ampulla of Vater, ovarian cancer, and urothelial carcinoma. This confirms the data according to which the most aggressive EMPD is the secondary form or that PVD is always associated with the presence of other oncological pathologies.

Despite the limitations of our study (limited number of patients retrospectively collected) we confirm that the diagnosis of Vulvar Paget's disease is often delayed, that recurrences are common independently of the presence of microinvasion and positive margins. Early recognition of secondary aggressive forms may reduce morbidity and mortality.

Different alternative treatments to surgery such as imiquimod therapy,^[18–20]^ as already mentioned, or photodynamic treatment are under investigation.^[18–20]^

In our case study, unlike others reported in the literature,^[3,4,21–23]^ there was only 1 case of a patient with a very aggressive disease with rapid spread and death. Furthermore, our study highlights the important role of plastic surgery.

Our study has no other novelties compared to other studies^[3,4,21–23]^ with a greater number of cases, but could serve to emphasize once again the delay with which this pathology is often diagnosed and the need to establish further therapeutic alternatives, considering the large number of relapses with repeated surgical treatments and consequent increase in morbidity due to repeated mutilations.

We hope that research will soon be carried out on large series of cases with the implementation of guidelines for this pathology which, although rare, may have a course with vulvar functional impairment, and negative surgical outcomes.

## Author contributions

**Investigation:** Rosalia Maria Rita Loiacono, Paola Traversi, Maria Assunta De Liso, Giulio Gargano, Anila Kardhashi, Roberta Francescato, Vera Loizzi, Marta Spinelli, Serena Lovascio, Maura Fiorito, Ondina Popescu.

**Supervision:** Leonardo Resta, Ettore Cicinelli, Gennaro Cormio.
